# Large-Scale Variation in Combined Impacts of Canopy Loss and Disturbance on Community Structure and Ecosystem Functioning

**DOI:** 10.1371/journal.pone.0066238

**Published:** 2013-06-14

**Authors:** Tasman P. Crowe, Mathieu Cusson, Fabio Bulleri, Dominique Davoult, Francisco Arenas, Rebecca Aspden, Lisandro Benedetti-Cecchi, Stanislao Bevilacqua, Irvine Davidson, Emma Defew, Simonetta Fraschetti, Claire Golléty, John N. Griffin, Kristjan Herkül, Jonne Kotta, Aline Migné, Markus Molis, Sophie K. Nicol, Laure M-L J. Noël, Isabel Sousa Pinto, Nelson Valdivia, Stefano Vaselli, Stuart R. Jenkins

**Affiliations:** 1 School of Biology and Environmental Science, University College Dublin, Dublin, Ireland; 2 Department of Biology, University of Pisa, Consorzio Interuniversitario per le Scienze del Mare, Pisa, Italy; 3 Département des sciences fondamentales, Université du Québec à Chicoutimi, Chicoutimi, Québec, Canada; 4 Université Pierre et Marie Curie Université Paris 06, Station Biologique de Roscoff, Roscoff, France; 5 Centre National de la Recherche Scientifique, Station Biologique de Roscoff, Roscoff, France; 6 Laboratory of Coastal Biodiversity, Centre of Marine and Environmental Research, University of Porto, Porto, Portugal; 7 Scottish Oceans Institute, University St Andrews, St Andrews, Fife, Scotland; 8 Department of Biological Environmental Science and Technology, Università del Salento, Consorzio Nazionale Interunversitario per le Scienze del Mare, Lecce, Italy; 9 Marine Biological Association, Plymouth, United Kingdom; 10 Estonian Marine Institute, University of Tartu, Tallinn, Estonia; 11 Section Functional Ecology, Biologische Anstalt Helgoland, Alfred-Wegener-Institute for Polar and Marine Research, Helgoland, Germany; 12 Instituto de Ciencias Marinas y Limnológicas, Laboratorio Costero de Recursos Acuáticos Calfuco, Universidad Austral de Chile, Campus Isla Teja, Valdivia, Chile; 13 School of Ocean Sciences, Bangor University, Menai Bridge, Anglesey, United Kingdom; 14 Department of Biosciences, Swansea University, Swansea, United Kingdom; University of British Columbia, Canada

## Abstract

Ecosystems are under pressure from multiple human disturbances whose impact may vary depending on environmental context. We experimentally evaluated variation in the separate and combined effects of the loss of a key functional group (canopy algae) and physical disturbance on rocky shore ecosystems at nine locations across Europe. Multivariate community structure was initially affected (during the first three to six months) at six locations but after 18 months, effects were apparent at only three. Loss of canopy caused increases in cover of non-canopy algae in the three locations in southern Europe and decreases in some northern locations. Measures of ecosystem functioning (community respiration, gross primary productivity, net primary productivity) were affected by loss of canopy at five of the six locations for which data were available. Short-term effects on community respiration were widespread, but effects were rare after 18 months. Functional changes corresponded with changes in community structure and/or species richness at most locations and times sampled, but no single aspect of biodiversity was an effective predictor of longer-term functional changes. Most ecosystems studied were able to compensate in functional terms for impacts caused by indiscriminate physical disturbance. The only consistent effect of disturbance was to increase cover of non-canopy species. Loss of canopy algae temporarily reduced community resistance to disturbance at only two locations and at two locations actually increased resistance. Resistance to disturbance-induced changes in gross primary productivity was reduced by loss of canopy algae at four locations. Location-specific variation in the effects of the same stressors argues for flexible frameworks for the management of marine environments. These results also highlight the need to analyse how species loss and other stressors combine and interact in different environmental contexts.

## Introduction

Ecosystems are threatened by a range of pressures and damage to their structure and functioning, which can have important consequences for society [1]. Although the effects of individual stressors on ecosystems have been widely studied, most are acted upon simultaneously by multiple stressors [2],[3], [4]. It is therefore critical that we improve our understanding of the ways in which effects of one stressor are modified by the action of others [5],[6]. Loss of biodiversity, for example through harvesting and habitat destruction, is a key threat to ecosystems [1]. It can be thought of as a stressor and is known to affect a range of ecosystem functions and properties, including productivity, respiration and stability [7],[8], [9]. In this context, a key aspect of stability is ‘resistance’, the capacity of a system to remain unchanged when disturbed, for example by physical stress [10], [11]. Certain species can buffer against large disturbances and therefore enhance resistance of communities to stressors. The loss of these key species, may therefore make ecosystems more susceptible to the increased levels of physical disturbance forecast as part of global climate change [12], but we cannot predict these effects with our current knowledge of most systems.

Although a substantial body of research is being accumulated, the generality of effects of loss of species for marine ecosystems is not well understood. We are developing some good understanding of effects of biodiversity loss at some ‘research hotspots’, such as seagrass and macroalgal habitats of Southeastern USA [13],[14], [15] and sedimentary shores of the Ythan estuary [16],[17], but for many other sites and systems there is no history of research and little basis for prediction. To develop a more general framework to predict effects of loss of biodiversity, we need information on spatial and temporal variation in experimental outcomes. This information is also essential for the implementation of environmental legislation which requires spatially defined action to conserve the functionality of marine ecosystems, such as the new EU Marine Framework Strategy Directive [18].

Fucoids and other canopy-forming macroalgae are recognised as key structural and functional elements of marine ecosystems at a wide range of locations [19], [20]. It would therefore be expected that their functional role could not easily be fulfilled by other species (*sensu* [21], [22]). Canopy algae are thought to be in worldwide decline and many local extinctions have been documented, particularly in Europe [19], [23]. Algal dominated ecosystems are extremely productive [24], exporting biomass and underpinning detrital food webs in coastal ecosystems. They are among the habitats most threatened by multiple stressors [25] and many will experience increasing physical stress under forecast climate change scenarios which include increased storminess [26]. Previous research has shown a range of responses to canopy removal, including replacement by grazers or turfs [27], [19], reductions in some understory species and/or invertebrate abundance and richness [19], [28], [29] or reductions in algal biomass and productivity [30]. Recovery of algal and invertebrate communities varies considerably and may take up to 4, 6 or even 12 years (e.g. [31], [32], [33]) The direction and rate of recovery depends on complex interactions among species and variation in local abiotic conditions and season that influence reproduction, dispersal, recruitment and growth [34], [31].

Intertidal rocky reefs are tractable model systems with a long history of valuable ecological research (e.g. [35]). Studies of ecosystem processes on intertidal rocky reefs, however, have been limited to some degree by technical challenges. Although photosynthetic and respiratory rates in rockpools can conveniently be measured as oxygen fluxes [36], [37], primary productivity on emersed rock has generally been assessed using proxies such as percentage algal cover and biomass accumulation (e.g. [38]). Recently, direct measurements of emersed CO_2_ fluxes have also proven successful in measuring the gross primary production and community respiration of shores dominated by macroalgae [39]. Indeed, intertidal macroalgae spend a substantial proportion of their time emersed and, although extreme light exposure and desiccation can affect productivity, most intertidal macroalgae still display high rates of carbon assimilation even after 30% to 60% water loss [40], ensuring a meaningful contribution of emersed productivity to total productivity in many cases [41].

This study examined variation in the separate and combined effects of the loss of key functional taxa (canopy algae) and of physical disturbance on rocky shore ecosystems. We used a field experiment to test whether the loss of canopy algae reduces physical protection for other species and thus reduces the capacity of rocky intertidal systems to resist physical disturbance, both in terms of community structure and ecosystem functioning, and whether its loss can be compensated for to any extent by other species in the assemblage. Variation in impacts of these stressors was assessed by replicating the experiment at nine locations across Europe and sampling it over an 18 month period. The locations spanned a latitudinal gradient from 56 to 40° North, included macro- and micro-tidal sites and encompassed considerable variation in algal community structure, from dense beds of brown fucoid algae in the northern sites to *Cystoseira* and low turfs in the Mediterranean and mixed small species on the coast of Portugal. Canopy may thus play different roles under these different circumstances, potentially causing variation in effects of its loss [c.f. 42].

## Methods

### Ethics Statement

No specific permits were required for St Andrews, Dublin, Plymouth, Porto or Roscoff. In each case, we confirm that the locations were not privately owned or protected and that the field studies did not involve endangered or protected species, nor were non-indigenous species introduced. For Livorno, the University of Pisa obtained all necessary permits for the described field studies from the Council of Livorno, Italy. For Lecce, the University of Salento obtained all necessary permits for the described field studies by the Marine Protected Area of Porto Cesareo (Lecce), Italy. On Helgoland, the research adhered to the legal requirements of the Schleswig-Holstein state act of 24 April 1981 (classification number 791-4-37) that declared the rocky shores below the high tide limit in Helgoland to be a nature reserve and allow ecologists to conduct and maintain manipulative experiments.

### Study Systems

**Figure 1:**
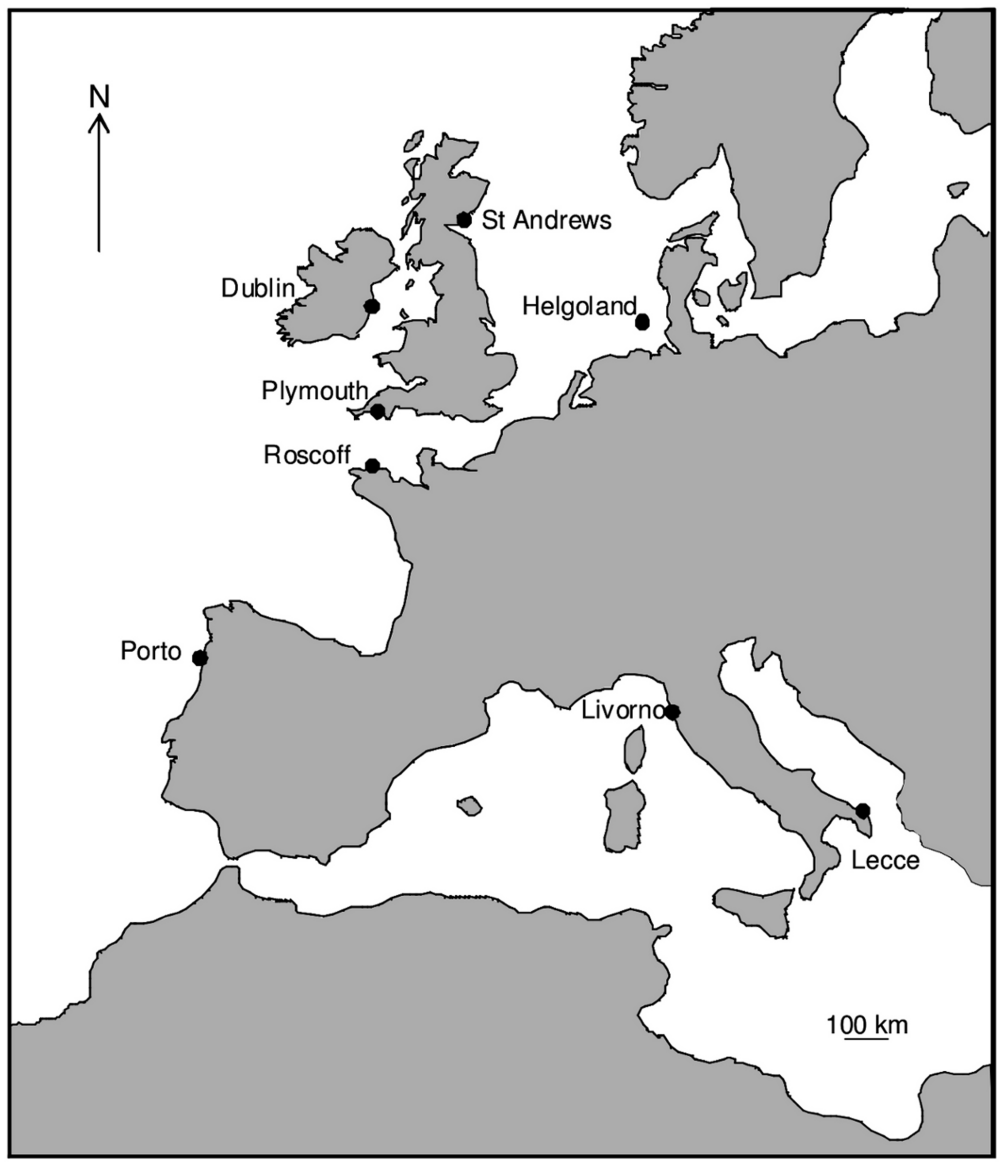
Map of study locations. Two locations were studied at Helgoland. In the text, Porto, Livorno and Lecce are referred to as southern locations and the other locations are considered to be northern.

Experiments were done on rocky shores at nine locations in Europe (Fig. 1, Table 1). Three were in southern Europe (Lecce and Livorno in the Mediterranean and Porto on the Atlantic coast of Portugal), the remainder were in northern Europe (Fig. 1, Table 1), including two on the island of Helgoland. The species making up the canopy varied among locations, but in each case were considerably larger than the other members of the algal assemblage and formed an extensive layer above them: at the northern locations it was *Fucus serratus*, in Lecce it was *Cystoseira amentacea*, in Livorno *Cystoseira compressa* and in Porto a mixture of small canopy species: *Mastocarpus stellatus*, *Chondrus crispus* and *Gigartina pistillata* (Table 1).

**Table 1:** Summary of details of study locations.

Location	Country	Position	Tidal range (m)	Shore level	Species of canopy algae
St Andrews	UK	56°19'59"N 2°46'19"W	5	Mid	*Fucus serratus*
Dublin	Ireland	53°31'27"N 6°4'49"W	5	Low	*Fucus serratus*
Helgoland 1	Germany	54° 11′N, 7° 53′E	2.4	Mid - Low	*Fucus serratu*s
Helgoland 2	Germany	54° 11′N, 7° 53′E	2.4	Mid-Low	*Fucus serratus*
Porto	Portugal	41°41′ N 8°51′W	4	Low	*Mastocarpus stellatus, Chondrus crispus, Gigartina pistillata*
Plymouth	UK	50°20.28′N 4°27.43′W	6	Low	*Fucus serratus*
Roscoff	France	48°43.84′N 3°59.27′ W	9	Mid-Low	*Fucus serratus*
Livorno	Italy	43°30′N, 10°20′E	0.3	Low	*Cystoseira compressa*
Lecce	Italy	40°13′N, 17°55′E	0.3	Low	*Cystoseira amentacea*

### Experimental Design and Manipulations

A factorial design was used, with the factors Canopy (removed (−), not removed (+)) and Disturbance (applied (+), not applied (−)). At each of the locations described above, 20 plots (30×30 cm) were selected in areas with >70% cover of canopy algae. Five plots were randomly assigned to each of four treatments: (i)+Canopy, − Disturbance; (ii)+Canopy,+Disturbance; (iii) -Canopy, − Disturbance; (iv) − Canopy,+Disturbance. In plots assigned to treatments involving canopy removal, all canopy algae were first removed by cutting carefully at the base of the holdfast with a knife. In plots assigned to treatments involving disturbance, disturbance was then applied. This treatment was designed to simulate the effects of severe physical disturbance such as that caused by the impact of waves and rocks during storms, for example. Pilot studies established the mean number of haphazard strokes of a scraping tool (chisel or similar) required to remove all biomass from a plot at each location. The disturbance applied to experimental treatments at each location was standardised as half of that number. Although no quantitative data were collected, the authors’ personal observations suggest that this tended to remove approximately 50% of the biomass from each plot. Canopy removals were maintained throughout the experiment (simulating an extended period of canopy loss); disturbance was only applied once (simulating a single extreme event). The experiment was initiated in Feb–Apr 2006 and maintained until Aug–Sept 2007.

### Sampling

#### Community structure

Quadrats (30×30 cm) were used to sample percentage cover of algae and sessile invertebrates and abundance of mobile invertebrates. Sampling was undertaken prior to manipulation and at intervals thereafter for 18 months. In this paper, findings from late summer 2006 (after 3–6 months depending on the location) and late summer 2007 (after 18 months) are reported, in order to account for short- and longer-term effects of our manipulation. Identification was done to the lowest taxonomic level possible in the field (usually species). Biomass was sampled destructively at the end of the experiment; biomass of different taxa was kept separate.

#### Ecosystem functioning

CO_2_ fluxes at the rock–air interface were measured using a benthic chamber connected to an infrared CO_2_ gas analyzer (LiCor Li- 800; LI-COR Inc., Lincoln, NE, USA), as described by Migné et al. [43]. The chamber consisted of a transparent Plexiglas dome and a 30×30 cm transparent Plexiglas base for a total volume of 18.3 to 18.9 L. An airtight seal between the chamber and the rock surface was achieved using a silicon joint neutral for CO_2_. Changes in CO_2_ mole fraction (ppm) were measured during 5–20 min incubations, depending on the system response, and data were recorded every 15 s with a data logger (LiCOr Li-1400; LI-COR Inc.). CO_2_ fluxes were calculated from the slope of CO_2_ concentration (µmol_CO2_.mol_ air_^−1^) against time (min). Results were then expressed in carbon units (mmolC.m^−2^.h^−1^) assuming a molar volume of 22.4 L.mol^−1^ at standard temperature and pressure. Measurements were performed under ambient light to assess the rate of benthic community net primary production (NPP) and in darkness, by covering the chamber with an opaque polyethene sheet, to assess benthic community respiration (CR). Before switching to the dark incubation, the dome was systematically opened to allow the system to return to ambient CO_2_ concentration. Benthic community gross primary production (GPP) was then calculated as the sum of NPP and CR. In the absence of information regarding the saturating irradiance of the communities during emersion (i.e. thalli more or less flattened in a multi-layer structure on the substratum), care was taken to perform the measurements with PAR (400–700 nm) above 300 µmolphotons.m^−2^.s^−1^, since saturating irradiance values for emersed intertidal algae have been measured in this range (e.g. [44], [45]). Working at or near saturating irradiance levels provides a good basis for comparisons between treatments and locations despite variations in irradiance. Measurements on quadrats from different treatments were done in random order to ensure that any variability during the emersion period would not confound differences among treatments. Inadequate light or rough sea conditions combined with limited tidal range prevented measurement of ecosystem functioning (CO_2_ fluxes) on one or more sampling occasions at a number of locations, notably Livorno and Lecce.

In testing effects of loss of canopy on ecosystem functioning, it was considered realistic to leave the canopy in place for functional measures in plots from which it had not been removed at the outset of the experiment. However, we recognise that the canopy itself may underpin any observed differences in functioning between plots from which it had been removed and plots in which it remained. To improve interpretation of functional differences that may have arisen due to changes in the remainder of the assemblage, additional measurements of CR were made after 18 months after having carefully removed the canopy algae from plots in which it had been left in place at the outset of the experiment. This allowed us to assess the extent to which loss of canopy could be compensated for (in terms of CR) by the remainder of the assemblage.

### Analyses

#### Community structure

In all analyses, the canopy algae that had been manipulated were excluded from the datasets. Multivariate community structure was visualised using non-metric Multi-Dimensional Scaling (nMDS) based on Bray-Curtis similarities and analysed using distance-based permutational multivariate analysis of variance (PERMANOVA [46], [47]). The factors were Location (random, orthogonal, 9 levels), Canopy (fixed, orthogonal, 2 levels) and Disturbance (fixed, orthogonal, 2 levels). Data were square root transformed to decrease the contribution of dominant species to the multivariate patterns. Each term in the analysis was tested by 999 random permutations of raw data. Separate analyses were done on data collected 3–6 months and 18 months after the initiation of the experiment.

Three-factor ANOVAs based on the model described above were used to analyse univariate data collected at each time. The number of replicates in a given analysis varied between 4 and 5, but analyses were always balanced. The following variables describing community structure were derived and analysed: taxon richness; evenness (on biomass sampled after 18 months only because data collected after 3–6 months comprised a mixture of cover and abundance values); total algal cover; cover of canopy forming algae; cover of non-canopy forming algae; total cover of sessile invertebrates; total abundance of mobile invertebrates, total biomass after 18 months. Cochran’s test was used to test for heterogeneity of variances and transformations were used to achieve homoscedasticity where appropriate. Post-hoc pooling of terms that were non-significant (*P*>0.25) enabled more powerful tests of remaining terms in the analysis of non-canopy algae after 18 months [48].

An impact of loss of canopy on the susceptibility of the system to physical disturbance (i.e. a change in resistance), was inferred from significant Canopy x Disturbance interactions in PERMANOVA or ANOVA. Specifically, a reduction in resistance caused by the loss of canopy would be inferred from a greater difference between disturbed and undisturbed treatments where canopy was absent than where it was present. This operational definition of resistance applies whether the impact of disturbance is negative or positive. Independent effects of canopy loss or disturbance were inferred from non-significant interactions combined with significant main effects for the terms Canopy and Disturbance respectively. Spatial variation in the impacts of these stressors was inferred from significant interactions involving Location, i.e. Location x Canopy, Location x Disturbance, Location x Canopy x Disturbance. Significant terms were further examined using pairwise comparisons (PERMANOVA) or Student Newman Keuls procedure (ANOVA) as appropriate.

### Ecosystem Functioning

The variables describing ecosystem functioning (GPP, NPP, CR) were each analysed using the same three factor ANOVA model and procedures described above. Separate analyses were done for data collected after 3–6 and 18 months. The number of replicates and locations varied depending on the availability of data, but all analyses were balanced. Two analyses were done for the 18 month sampling period: one on measurements made prior to the removal of canopy algae from plots in which they were present and one on measurements made after its removal (see Sampling section above). This enabled us to account for the influence of the canopy algae itself on functional measures at this final sampling date.

## Results

At most locations, between 20 and 35 algal species were found; exceptions were Plymouth (50 species) and Porto (71 species). Richness of animal taxa was low in Porto (5 species), high in Roscoff (50 species) and ranged between 15 and 27 species at the other locations.

### Effects of Loss of Canopy on Community Structure

**Figure 2:**
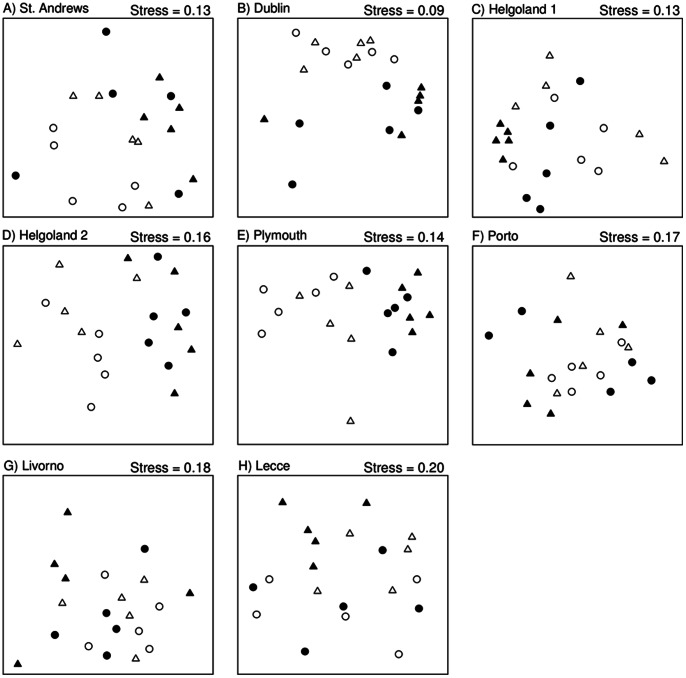
nMDS illustrating the effects of canopy removal (filled symbols = + canopy present; hollow symbols = − canopy) and application of mechanical disturbance (circle = + disturbance; triangle = − disturbance) on assemblages 3–6 months after the start of the experiment, separately for each study location. Data were square root transformed. (n = 5). Data for Roscoff are not included because only presence-absence and biomass data were recorded there.

Three to six months after the start of the experiment, loss of canopy had affected multivariate community structure at all of the locations sampled in the north of Europe and at Lecce in the south. At Dublin, Helgoland 2, Plymouth and Lecce the difference was consistent regardless of disturbance; at St Andrews and Helgoland 1, the loss of canopy affected community structure only in the absence of disturbance (Table 2, Fig. 2). There were no effects of loss of canopy at Porto or Livorno (Fig. 2, Table 2). Eighteen months after the start of the experiment, differences in overall multivariate community structure remained only at Dublin, Plymouth and Livorno (Table 3).

**Table 2:** Summary of impacts of canopy loss and disturbance on ecosystem structure after 3–6 months.

	Community			Richness			Canopy (%)			Non-canopy (%)			Sessile (%)			Mobile (No.)		
	C	D	CD	C	D	CD	C	D	CD	C	D	CD	C	D	CD	C	D	CD
St Andrews	+^1^			**−**	**−**					**−**	**−**							
Dublin	+						**−**			+			**−**					
Helgo 1	+^1^		+				+			**−**	**−**							
Helgo 2	+									**−**								
Plymouth	+		**−**															
Roscoff	na	na	na	**−**			na	na	na	na	na	na	na	na	na	na	na	na
Porto										+								
Livorno				+						+								
Lecce	+^1^		+		**−**													

C = Canopy, D = Disturbance, CD = Canopy x Disturbance interaction. For community analyses (PERMANOVA), a ‘+’ symbol indicates any significant difference in community structure. For univariate measures, a ‘+’ symbol indicates a significant positive effect of applying the treatment (e.g. removal of canopy increases taxon richness), ‘**−**’ symbol indicates a significant negative effect of applying the treatment (e.g. disturbance reduces taxon richness). For CD, a ‘**−**’ symbol indicates that loss of canopy reduced stability (i.e. increased impact of disturbance) and a ‘+’ symbol indicates that loss of canopy increased stability (i.e. reduced impact of disturbance). In each case, no symbol indicates no significant result and ‘na’ indicates data unavailable. ‘Sessile’ refers to sessile invertebrates, ‘%’ refers to percentage cover, ‘Mobile’ refers to mobile invertebrates, ‘No.’ refers to number per quadrat. At Roscoff only presence-absence data were recorded, so only richness was analysed.

1 in absence of disturbance only.

**Table 3:** Summary of impacts of canopy loss and disturbance on ecosystem structure after 18 months.

	Community			Richness			Evenness			Canopy (%)			Non-canopy (%)			Sessile (%)			Mobile (No.)		
	C	D	CD	C	D	CD	C	D	CD	C	D	CD	C	D	CD	C	D	CD	C	D	CD
St Andrews				**−**									**−**	+					**−**		
Dublin	+								+					+		**−**					
Helgo 1														+							
Helgo 2														+							
Plymouth	+			**−**										+							
Roscoff	na	na	na							na	na	na	na	na	na	na	na	na	na	na	na
Porto				+									+	+							
Livorno	+												+	+							
Lecce														+							

C = Canopy, D = Disturbance, CD = Canopy x Disturbance interaction. For community analyses (PERMANOVA), a ‘+’ symbol indicates any significant difference in community structure. For univariate analyses (all others), a ‘+’ symbol indicates a significant positive effect of applying the treatment (e.g. removal of canopy increases taxon richness), ‘**−’** symbol indicates a significant negative effect of applying the treatment (e.g. disturbance reduces taxon richness). For CD, a ‘**−**’ symbol indicates that loss of canopy reduced stability (i.e. increased impact of disturbance) and a ‘+’ symbol indicates that loss of canopy increased stability (i.e. reduced impact of disturbance). In each case, no symbol indicates no significant effects and ‘na’ indicates data unavailable. ‘Sessile’ refers to sessile invertebrates, ‘%’ refers to percentage cover, ‘Mobile’ refers to mobile invertebrates, ‘No.’ refers to number per quadrat. At Roscoff only presence-absence and biomass data were recorded, so only richness and evenness were analysed.

**Figure 3:**
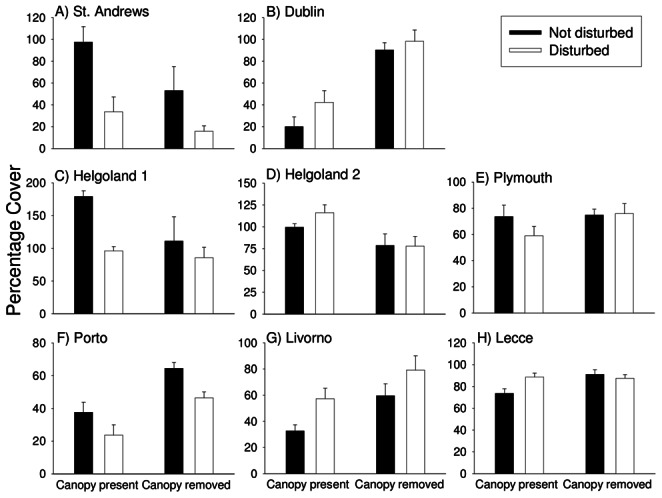
Effects of canopy removal and disturbance on the percentage cover of non-canopy macroalgae 3–6 months after the start of the experiment. Data are mean+SE (*n* = 5). Data for Roscoff are not included because percentage covers were not recorded there.

**Figure 4:**
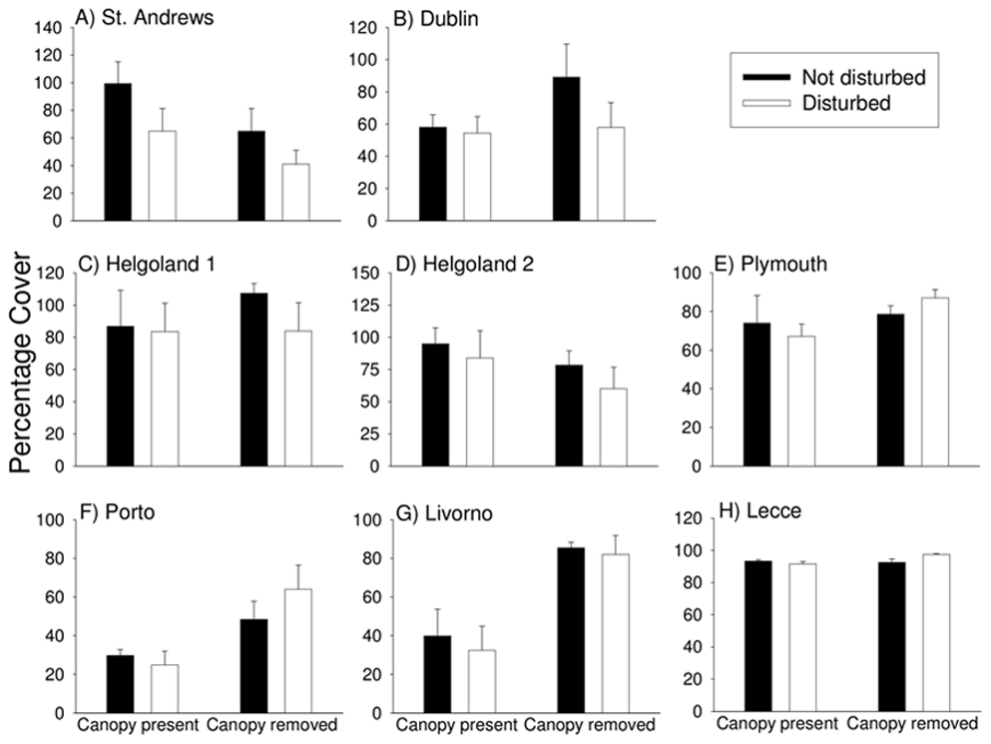
Effects of canopy removal and disturbance on the percentage cover of non-canopy macroalgae 18 months after the start of the experiment. Data are mean+SE (*n* = 5). Data for Roscoff are not included because percentage covers were not recorded there.

There were few significant effects of canopy loss on univariate measures of community structure. Three to six months after initiation of the experiment, taxon richness was affected only in Livorno, where it increased, and Roscoff and St Andrews, where it decreased (Table 2). After 18 months, richness was negatively affected at St Andrews and Plymouth and positively affected in Livorno (Table 3). Evenness was not affected at any location (Table 3). The group most frequently affected by canopy removal was non-canopy algae, but effects varied among locations (Table 3). After 3–6 months, negative impacts were recorded in Helgoland and St Andrews; positive effects were seen at Livorno, Porto and Dublin (Fig. 3, Table 2). These patterns were the same for total algal cover (Table 4). After 18 months, positive impacts were still apparent for non-canopy forms and for total algal cover at Livorno and Porto and negative impacts remained only at St Andrews (Fig. 4, Tables 3, 5). Sessile invertebrates were less common where canopy had been removed in Dublin both after 3–6 months and at the end of the experiment (Tables 2, 3). The same patterns were observed for mobile invertebrates in St Andrews at the end of the experiment (Table 3).

**Table 4:** Summary of impacts of canopy loss and disturbance on aspects of ecosystem structure and functioning after 3–6 months.

	Total cover			GPP			NPP			CR		
	C	D	CD	C	D	CD	C	D	CD	C	D	CD
St Andrews	**−**	+		**−**						**−**		
Dublin	+			**−** ^1^		+	**−**			**−**		
Helgo 1	**−**	+		**−**			**−**			**−**		
Helgo 2	**−**			na	na	na	na	na	na	na	na	na
Plymouth				**−**			**−**			**−**		
Roscoff	na	na	na	**−**			**−**			**−**		
Porto	+											
Livorno	+			na	na	na	na	na	na	na	na	na
Lecce				na	na	na	na	na	na	na	na	na

C = Canopy, D = Disturbance, CD = Canopy x Disturbance interaction. For C & D, a ‘+’ symbol indicates a significant positive effect of applying the treatment (e.g. removal of canopy increases the value of the response variable), ‘**−’** symbol indicates a significant negative effect of applying the treatment (e.g. disturbance reduces the value of the response variable). For CD, a ‘**−**’ symbol indicates that loss of canopy reduced stability (ie increased impact of disturbance) and a ‘+’ symbol indicates that loss of canopy increased stability (ie reduced impact of disturbance). In each case, no symbol indicates no significant effect and ‘na’ indicates data unavailable. ‘Total cover’ refers to total algal cover, ‘GPP’ refers to Gross Primary Productivity, ‘NPP refers to Net Primary Productivity and CR refers to ‘Community Respiration’.

1 only in the absence of disturbance.

**Table 5:** Summary of impacts of canopy loss and disturbance on aspects of ecosystem structure and functioning after 18 months.

	Total cover			Total biomass			GPP			NPP			CR			CR (− canopy)		
	C	D	CD	C	D	CD	C	D	CD	C	D	CD	C	D	CD	C	D	CD
St Andrews	**−**						**−**		**−**	**−**			−					
Dublin				+			na	na	na	na	na	na				na	na	na
Helgo 1							na	na	na	na	na	na	−				+	
Helgo 2				na	na	na	na	na	na	na	na	na	na	na	na	na	na	na
Plymouth				−			−		−	−						+		
Roscoff	na	na	na	−			−		−	−			−					
Porto	+								−								+	
Livorno	+						na	na	na	na	na	na	na	na	na	na	na	na
Lecce							na	na	na	na	na	na	na	na	na	na	na	na

C = Canopy, D = Disturbance, CD = Canopy x Disturbance interaction. For C & D, a ‘+’ symbol indicates a significant positive effect of applying the treatment (e.g. removal of canopy increases the value of the response variable), ‘−’ symbol indicates a significant negative effect of applying the treatment (e.g. disturbance reduces the value of the response variable). For CD, a ‘−’ symbol indicates that loss of canopy reduced stability (i.e. increased impact of disturbance) and a ‘+’ symbol indicates that loss of canopy increased stability (i.e. reduced impact of disturbance). In each case, no symbol indicates no significant effect and ‘na’ indicates data unavailable. ‘Total cover’ refers to total algal cover, ‘GPP’ refers to Gross Primary Productivity, ‘NPP refers to Net Primary Productivity and CR refers to ‘Community Respiration’.

### Effects of Loss of Canopy on Ecosystem Functioning

**Figure 5:**
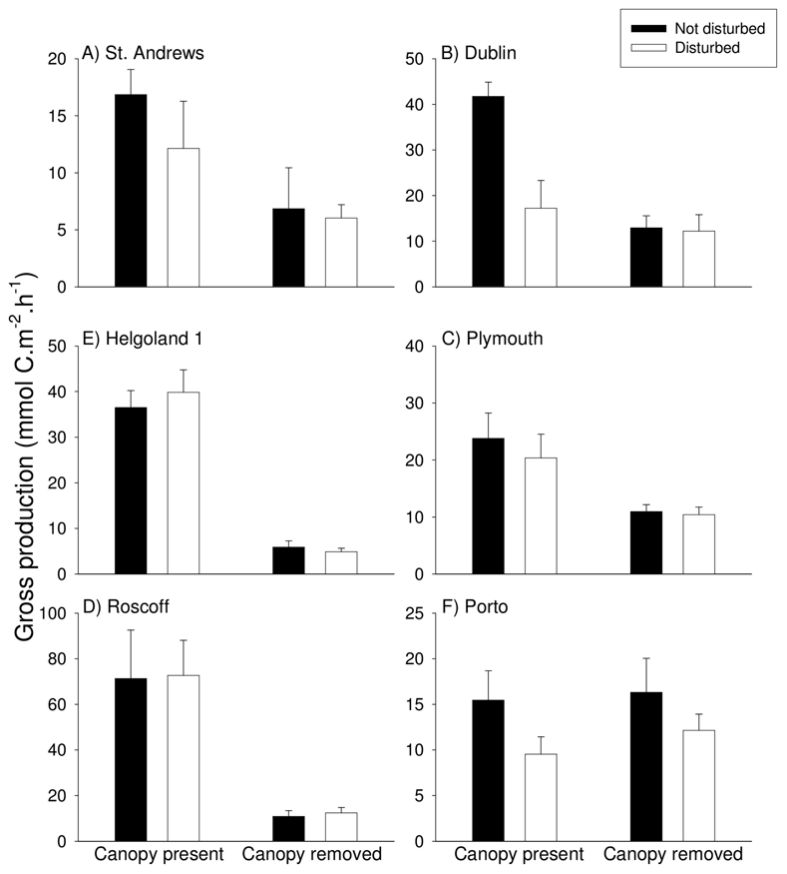
Effects of canopy removal and disturbance on gross primary productivity of assemblages 3–6 months after the start of the experiment. Data are mean+SE (*n* = 5 for A, C, E; *n* = 3 for B; *n* = 4 for D and F:).

**Figure 6:**
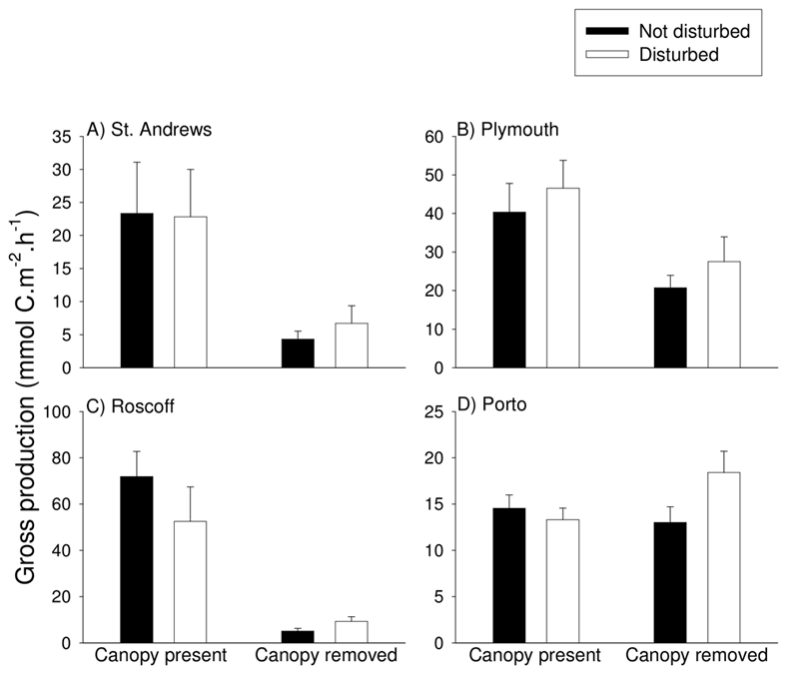
Effects of canopy removal and disturbance on gross primary productivity of assemblages 18 months after the start of the experiment. Data are mean+SE (*n* = 5 for A B and C; *n* = 3 for D).

After 3–6 months, loss of canopy reduced GPP at five of six locations (Fig. 5, Table 4). There was no effect in Porto (Fig. 5, Table 4). After 18 months, GPP could only be measured at four locations. At the four locations sampled on both occasions, findings were consistent with those after 3–6 months (Fig. 6, Table 5). NPP was also reduced at four of six locations after 3–6 months. Findings were consistent with those for GPP, except at Dublin and St Andrews (Table 4). Findings for NPP after 18 months were consistent with those for GPP (Table 5).

**Figure 7:**
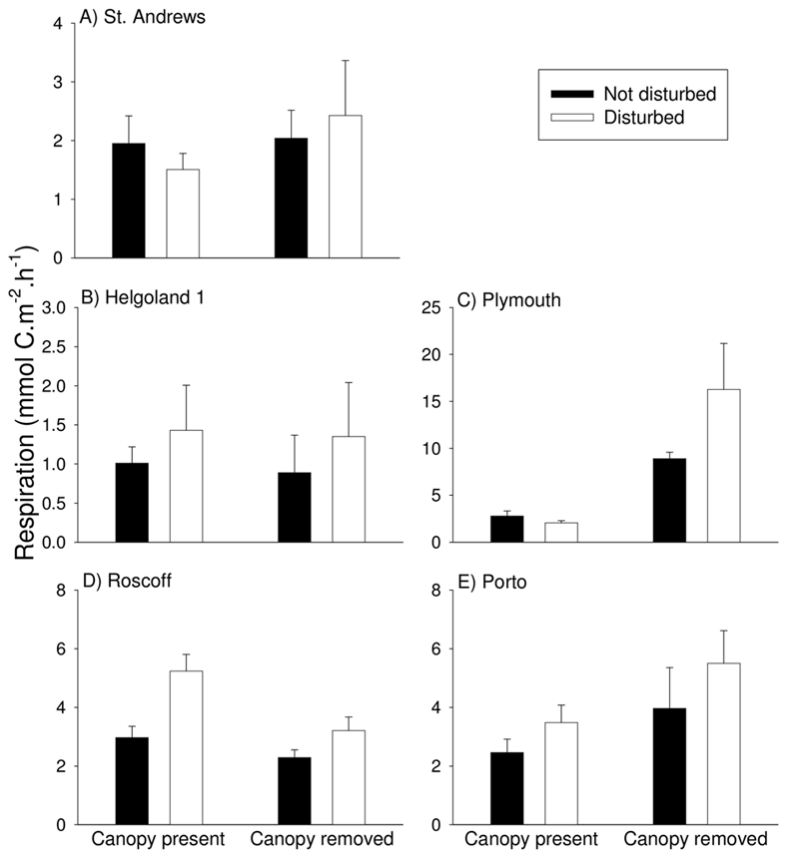
Effects of canopy removal and disturbance on community respiration of assemblages (excluding the contribution of the canopy species manipulated) 18 months after the start of the experiment. Data are mean+SE (*n* = 5 except for B and E: *n* = 3).

After 3–6 months, CR was reduced by loss of canopy at five of six locations for which data were available, all of them in the north of Europe (Table 4). After 18 months, CR was only affected by loss of canopy at Helgoland 1, Roscoff and St. Andrews of the six locations sampled (Table 5). At three of the six locations, therefore, the remainder of the assemblage was able to compensate for the loss of canopy in terms of CR. In fact, when the canopy algae themselves were removed from plots in which they had not been experimentally removed at the outset, no negative impacts of loss of canopy on CR of the remaining assemblage were detectable at any location (Fig. 7, Table 5). In Plymouth, comparisons after removing canopy algae from all plots showed an increase in CR in plots from which canopy had been removed at the outset (Table 5), again suggesting a high level of compensation by non-canopy species.

### Correspondence between Effects on Structure and Functioning

After 3–6 months, there was good correspondence between impacts of canopy loss on community structure and changes in functioning. At all five of the locations for which the comparison could be made, changes (or lack thereof) in multivariate community structure were matched by equivalent changes (or lack thereof) in some aspect of functioning (Table 6). At this stage, changes in taxon richness corresponded with changes in functioning at two locations (St Andrews and Porto) and changes in other measures corresponded with changes in functioning at three of the five locations (Table 6). After 18 months, correspondence between changes in multivariate community structure and changes in functioning had broken down – differences in community structure corresponded with differences in functioning at only two of the five locations (Table 6). At this stage, however, correspondence between effects on species richness and effects on functioning arose at three of the five locations (Table 6).

**Table 6:** Correspondence between impacts of canopy loss on ecosystem structure and functioning 3–6 and 18 months after initiation of the experiment (based on Tables 1–4).

	After 3–6 months				After 18 months			
*Impact on:*	Structure			Functioning	Structure			Functioning
	MV	Rich	Any		MV	Rich	Any	
St Andrews	•	•	•	•	○	•	•	•
Dublin	•	○	•	•	•	○	•	○
Helgo 1	•	○	•	•	○	○	○	•
Plymouth	•	○	○	•	•	•	○	•
Porto	○	○	•	○	○	•	•	○

Ecosystem structure is considered in terms of multivariate community structure ( = MV), species richness ( = Rich) and any of the other univariate measures ( = Any). • = significant result, ○ = non-significant result. A ‘•’ in the ‘Functioning’ column indicates a significant result in any of the three measures of functioning. Correspondence between change in functioning and change in an aspect of structure is indicated by the same symbols occurring in respective columns. Shown are all locations for which both data sets are available for both sampling times.

### Effects of Disturbance on Structure and Functioning

Disturbance had no independent effect on multivariate community structure at any location after either 3–6 or 18 months (Fig. 2, Tables 2, 3). Disturbance led to a reduction in taxon richness only at Lecce and St Andrews and that effect was no longer apparent 18 months after the start of the experiment (Tables 2, 3). The only taxa for which main effects of disturbance were apparent were non-canopy algae. After 3–6 months, their cover was reduced by disturbance at St Andrews and Helgoland 1 (Fig. 3, Table 2). After 18 months, there was an overall increase in non-canopy algae on average in disturbed plots compared with undisturbed plots, regardless of the presence or absence of canopy (Fig. 4, Table 3– a significant main effect of Disturbance).

Disturbance did not affect any measure of ecosystem functioning independently of the influence of canopy (Tables 4, 5). An independent effect of disturbance was only detected for two locations (Helgoland 1 and Porto) in analyses of CR done after canopy had been removed from all plots at the end of the experiment (Table 5).

### Effects of Loss of Canopy on Resistance to Disturbance

At only one location (Plymouth) did the loss of canopy cause an increase in the difference in multivariate community structure between disturbed and undisturbed plots compared to plots with canopy present (i.e. a reduction in the resistance of the community to disturbance); this effect was apparent only after 3–6 months (Fig. 2, Table 2– significant CxD interaction). At Helgoland 1 and Lecce, community structure was actually more resistant to disturbance in the absence of canopy, but only after 3–6 months (Fig. 2, Table 2). Loss of canopy did not affect resistance in any univariate measures of community structure, except for causing increased resistance of evenness at Dublin after 18 months (Tables 2, 3). In terms of ecosystem functioning, however, the resistance of GPP was reduced after 18 months (i.e. changes (increases) in GPP were caused by disturbance in the absence of canopy, but not in the presence of canopy) at all four of the locations for which measurements were made (Porto, Roscoff, Plymouth and St. Andrews; Fig. 6, Table 5). There was a positive effect of canopy loss on resistance of GPP at Dublin after 3–6 months (Fig. 5., Table 4), but no effects on resistance of NPP or CR at either time (Tables 4, 5).

## Discussion

Loss of apparently important species did not always affect the structure of European rocky shore ecosystems. Canopy shades organisms and reduces the impact of physical and biological factors, thereby facilitating associated species and maintaining high levels of local diversity [49]. Thus, loss of these species was expected to result in important changes in community structure, for example the replacement of fragile red perennial species by ephemeral green algae [20]. Multivariate community structure (based on all individual taxa identified) was initially affected at all of the northern locations sampled and one southern location, but these effects were detectable at only three locations after 18 months. The main impacts on community structure were manifested as effects on aggregated non-canopy algae, with a tendency for increased cover in the absence of canopy in southern locations and decreased cover in the absence of canopy in some northern locations. These apparent latitudinal trends may be linked with regional differences in the identity and morphology of canopy species and climatic conditions. For example, at some southern locations, such as Livorno, canopy algae (*C. compressa*) had short fronds and large bases and did not develop tall canopies. In these locations, negative effects of the canopy on understory algae, due to pre-emption of the substratum, likely outweighed positive ones due to amelioration of physical conditions (see also [42], [50]). Variation may also have arisen due to differences in tidal range and tidal elevation at experimental locations (Table 1).

Loss of canopy had significant impacts on ecosystem functioning at most but not all of the locations studied. Production of intertidal algal beds when emersed may not be as great as production when immersed (e.g. [51], [52]), although in some cases its contribution can be considerable (e.g. [53]) or even equivalent [54]. The intention here, however, was not to capture total productivity, but to use a practicable measure of ecosystem functioning to draw direct comparisons among locations about effects of experimental canopy loss and disturbance. In common with other findings (e.g. [55]), the consequences of biodiversity loss for ecosystem functioning depended on which function was measured. Here, impacts on NPP and GPP were widespread, generally similar and were broadly consistent in the short- and longer-term, despite considerable variation in community structure and environmental context. Porto was the only location for which no differences in NPP or GPP were attributable to canopy loss after 18 months. Canopy species were smaller at Porto than at the other intertidal locations and perhaps more easily replaced in functional terms by other species. The large canopy of brown fucoid algae of the northern European rocky shores studied here appears to be generally irreplaceable as a contributor to NPP and GPP. CR, on the other hand, was strongly affected in the short-term at almost all locations where it was sampled (the exception again being Porto), but differences caused by canopy loss were rare after 18 months. In terms of CR, the remainder of the assemblage was able to compensate for the loss of canopy species within a comparatively short period of time at all locations. This was confirmed by the final measurement of CR that was made after having removed canopy from all plots so that comparisons were based solely on the remaining assemblage.

The counterintuitive finding that GPP increased in response to a disturbance that removed algal biomass can potentially be explained by the fact that perennial species do not recover as quickly as ephemeral species from physical disturbance. Ephemeral species, particularly green algae such as *Ulva intestinalis*, grew extensively on disturbed plots. Given that ephemeral species have higher growth rates and per tissue photosynthetic production than perennial species, an increased prevalence of ephemeral species would thus have increased the overall GPP of the community and their rapid degradation would also have increased community respiration.

Although there was spatial and temporal variation, functional changes due to canopy loss corresponded with changes in multivariate community structure and/or species richness at almost all locations and times, providing evidence of a link between changes in biodiversity (caused by loss of canopy species) and changes in functioning. No single aspect of taxonomic biodiversity was an effective predictor of longer term functional changes, however. Changes in multivariate community structure corresponded consistently with changes in functioning in the short-term (after 3–6 months), but not after 18 months. Changes in taxon richness, albeit minor, did not correspond consistently with changes in functioning at either sampling time, suggesting further support for the importance of identity effects in marine ecosystems [38], [56], [8] and/or the need for alternative metrics of biodiversity, such as those based on functional traits [57], [58], [59]. In contrast to some recent findings [60] [61], which found stronger effects of diversity in intertidal systems after 18 and 24–36 months respectively (and see [62]), there was no evidence that the link between biodiversity and ecosystem functioning became stronger over longer periods of time. In the current study, the link between structural and functional changes was less apparent after 18 months than after 3–6 months.

Impacts of experimental disturbances varied to some extent, but most rocky shore ecosystems studied were resistant to substantial physical impact both in structural and functional terms. The only consistent effect of disturbance was to encourage the growth of non-canopy species (in plots with and without canopy). There is ample evidence indicating that opportunistic algal forms (e.g. filamentous) can readily colonize space made available by disturbance and loss of other species [19], [63]. The disturbance applied was severe but indiscriminate - all species were equally likely to be dislodged - so it is perhaps not surprising that in this case overall community structure was comparatively unaffected. Different outcomes may be expected if different disturbances are applied. However, the lack of differences in functioning between disturbed and undisturbed plots is surprising, particularly in the short-term, when there must have been substantial differences in total biomass between disturbed and undisturbed plots, indicating that community level responses can be very complex and likely influenced by the identity of the species involved. The implication of this result is that the post-disturbance assemblage was able to compensate in functional terms for the loss of biomass of other members of the assemblage [64], [65].

Loss of canopy rarely reduced the resistance of community structure in response to physical disturbance (only at Plymouth and only after 3–6 months) and in some cases actually increased resistance to disturbance of aspects of community structure (Helgoland 1 and Lecce). Although resistance of NPP and CR were not significantly affected by loss of canopy, resistance of GPP was reduced after 18 months at three of the four locations at which it was measured. This is an important finding as it constitutes empirical evidence that reliability of ecosystem services to society may be impacted by loss of individual functional groups such as canopy algae and reinforces the need to consider a range of ecosystem functions in BEF manipulations [55]. Multiple stressors acting simultaneously have the potential to interact, causing changes that are not predictable from knowledge of independent effects of single stressors (‘ecological surprises’ sensu Paine et al. [2]) creating a high degree of uncertainty in predictive models [66]. For most locations in the current study, effects of loss of a key functional group and disturbance acted independently rather than interactively. The independent effects observed here have also been shown in recent manipulations of stress from sedimentation and nutrients [67], [68]. If interactive effects of multiple stressors are found to be rare, predictions of combined impacts would be comparatively straightforward. It should be noted that the stressors applied here (canopy loss and physical disturbance) may effectively influence the system in similar ways and as such may be less likely to interact than stressors which act very differently. Research into combined effects of multiple stressors is sparse compared to research into effects of individual stressors studied in isolation [5], [6]. More experiments manipulating more than one stressor are needed if we are to predict consequences for marine ecosystems of the combined influence of local and global environmental changes.

This is one of the first experimental studies to assess large scale variation in impacts of loss of an important functional group on the functioning of marine ecosystems. Using a novel approach to *in situ* measurement of functional response variables (adapted from Migné et al. [43]), it has shown widespread but not universal effects of loss of a functional group that would generally have been assumed to play a major role in driving the structure and functioning of rocky shore ecosystems (although we recognise that its influence on functional responses has not been fully characterised as we did not measure immersed as well as emersed production). It has also revealed a remarkable degree of resistance to impacts of substantial physical disturbance and a perhaps surprising lack of effect of loss of canopy species on the capacity of rocky shore ecosystems to resist disturbance. Another key finding from this study is the strong link between changes in community structure and changes in functioning in the short-term and its breakdown in the longer term, at which time persistent functional changes were more prevalent than persistent structural changes. Such widely replicated research is essential for the development of a more general framework to predict effects of loss of biodiversity on ecosystem functioning. It is also required to inform the development of management plans that are tailored to specific regions and locations in order to maximise their effectiveness (e.g. [18]). The experiments were field based and realistic, but were quite simple so that they could be repeated at comparatively low cost. More detailed analyses of community dynamics in experiments at two of our study areas show consistent results at two locations separated by about 1 and 25 km in Helgoland, Germany [69] and in Portugal [70], respectively. Buffer effects of the canopy against disturbance were observed at both locations in Portugal [70], while effects of canopy removal strengthened asynchrony in populations and reduced community respiration at both locations in Helgoland [69]. In common with the current study, Boyer et al. [71] recently found a considerable degree of variation in the effects of species richness on algal biomass production in four mesocosms and four field studies at locations within 20 km of each other. They did show, however, that similar mechanisms were operating in most environmental contexts.

Although meta-analyses of existing datasets are revealing some generalities, they also show a very high degree of variation in responses to biodiversity loss in different systems [72], [56], [73], [74]. Among the next important tasks in developing a coherent view of consequences of biodiversity loss is to identify general patterns in the circumstances (e.g. environmental context, initial ecosystem structure) under which particular outcomes of biodiversity loss can be expected. Both laboratory and field based research will be of value in this task [75], [71], [76], although long-term field experiments are more likely to yield directly applicable findings [60], [61], [62]. With the mechanistic understanding derived from such research, it should be possible to develop and refine models to predict consequences of realistic scenarios of biodiversity loss in contexts of varying initial ecosystem structure and under the influence of multiple stressors [77], [78].
